# Novel drugs that target the metabolic reprogramming in renal cell cancer

**DOI:** 10.1186/s40170-016-0154-8

**Published:** 2016-07-13

**Authors:** Johannes C. van der Mijn, David J. Panka, Andrew K. Geissler, Henk. M. Verheul, James W. Mier

**Affiliations:** Department of Hematology/Oncology, Beth Israel Deaconess Medical Center and Harvard Medical School, 330 Brookline Ave, Boston, MA 02215 USA; Department of Medical Oncology, VU University Medical Center, De Boelelaan 1117, 1081 HV Amsterdam, The Netherlands; Department of Internal Medicine, OLVG; Jan van Tooropstraat 164, 1061 AE Amsterdam, The Netherlands

**Keywords:** Warburg, Renal cell cancer, HIF, MYC, Glutamine

## Abstract

Molecular profiling studies of tumor tissue from patients with clear cell renal cell cancer (ccRCC) have revealed extensive metabolic reprogramming in this disease. Associations were found between metabolic reprogramming, histopathologic Fuhrman grade, and overall survival of patients. Large-scale genomics, proteomics, and metabolomic analyses have been performed to identify the molecular players in this process. Genes involved in glycolysis, the pentose phosphate pathway, glutamine metabolism, and lipogenesis were found to be upregulated in renal cell cancer (RCC) specimens as compared to normal tissue. Preclinical research indicates that mutations in VHL, FBP1, and the PI3K-AKT-mTOR pathway drives aerobic glycolysis through transcriptional activation of the hypoxia-inducible factors (HIF). Mechanistic studies revealed glutamine as an important source for de novo fatty acid synthesis through reductive carboxylation. Amplification of MYC drives reductive carboxylation. In this review, we present a detailed overview of the metabolic changes in RCC in conjunction with potential novel therapeutics. We discuss preclinical studies that have investigated targeted agents that interfere with various aspects of tumor cell metabolism and emphasize their impact specifically on glycolysis, lipogenesis, and tumor growth. Furthermore, we describe a number of phase 1 and 2 clinical trials that have been conducted with these agents.

## Background

Renal cell cancer (RCC) is an aggressive type of cancer that arises from the proximal renal tubular epithelium of the kidneys. It occurs as sporadic cancer as well as part of hereditary cancer syndromes. RCC accounts for ~80 % of all sporadic kidney cancers and has displayed a rising incidence over the last decades [1, 2]. Different histological subvariants of RCC can be recognized with “clear cell renal cell cancer” representing 88 % in contemporary pathological series [3]. Papillary and chromophobe cancer are other subtypes of RCC. Identification of the von Hippel-Lindau (VHL) gene, as most frequently deleted gene in RCC cells, accelerated development of vascular endothelial growth factor (VEGF)-targeted therapy for treatment of the disease. Silencing of VHL, resulting in accumulation of the hypoxia-inducible factor (HIF) transcription factors, induces elevated levels of VEGF in tumor cells. Upregulation of VEGF has been associated with the rich vascularization of tumors that is typically seen in tumors of patients with RCC. Five VEGF-targeted drugs (sunitinib, pazopanib, sorafenib, axitinib, and bevacizumab) are registered as therapeutic agent for treatment of RCC. Despite an increasing number of therapeutic options, the prognosis of patients with advanced RCC remains dismal. Locally advanced or metastatic RCC cannot be cured with current treatments.

A large retrospective cohort study was performed by “The Cancer Genome Atlas” (TCGA) consortium among patients with RCC [4]. Upregulation of genes involved in glycolysis was found, while genes, known to play a role in the tricarboxylic acid (TCA) cycle, were found to be downregulated [4, 5]. The altered pattern in glucose metabolism in tumors has first been described by the German physiologist Otto Warburg as aerobic glycolysis [6]. This phenomenon, nowadays frequently referred to as “the Warburg effect,” has been found in an important subset of the patients with RCC and was associated with a poor prognostic outcome. In this review, we will provide a detailed overview of the genetic changes that cause the Warburg effect, in conjunction with therapeutic agents that may interfere with this process.

Besides changes in glucose metabolism, numerous other metabolic changes have been detected in RCC specimens and include amino acid and lipid metabolism. Arginine, tryptophan, and glutamine are examples of amino acids with altered dynamics. Genetic defects in cancer cells lead to increased import of these amino acids from the extracellular space. Furthermore, upregulation was found of genes involved in de novo fatty acid synthesis (lipogenesis) by TCGA [4]. Upregulation of lipogenesis and intracellular storage of lipid droplets causes the typical histopathological “clear cell” phenotype in RCC (clear cell renal cell cancer (ccRCC)) [7]. Preclinical research indicated that increased lipid storage also alleviates endoplasmic reticulum (ER) stress [7]. Dependence on extracellular amino acid supplies and upregulation of lipogenesis were shown to create vulnerabilities that can potentially be exploited by therapeutic agents such as Peg-Arg-1 and 5-(tetradecycloxy)-2-furoic acid (TOFA) [8, 9].

Tumors with nutrient metabolism reprogramming carry a poor prognostic outcome, which suggests potential of this process as a drug target. The metabolic changes are a common molecular feature of advanced RCC tumors. The frequency of genetic changes is of importance for the selection of potential therapeutic targets. The fundamental idea of anticancer drugs is to target molecules and cellular processes that are essential for tumor cell survival and less important to non-proliferating normal cells. Selective pressure due to survival advantage of certain genetic defects may give enrichment of those alterations in tumors. A higher frequency of these genetic defects in patient series also creates a larger platform for drugs that specifically interfere with it. The VHL-HIF-VEGF axis as most frequently activated pathway in RCC and therapeutic target is here an exemplary case. The frequency of the metabolic reprogramming may render RCC a suitable disease for the investigation of potential novel therapeutic agents that target tumor metabolism.

## Part I. Metabolic pathways with altered activity in human RCC

### Results from molecular profiling studies of human RCC specimens

**Fig. 1:**
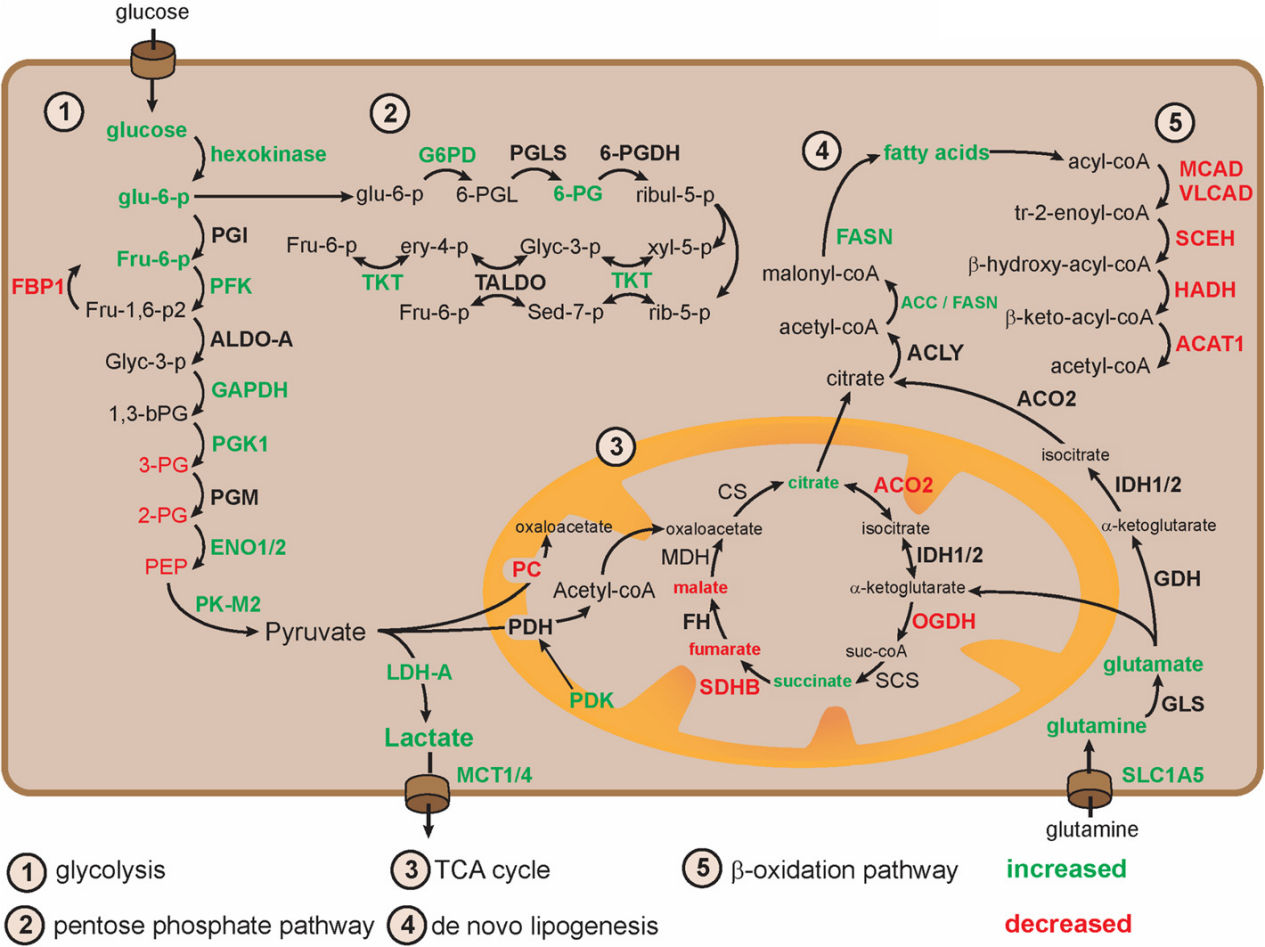
An overview of the molecular players in the metabolism of glucose, fatty acids, and glutamine. Pathways were divided in five different sections based on recognition as different entity in biochemical literature. Molecules labeled green were found to be upregulated in RCC specimens, while red molecules were downregulated

Frequent mutation of components in the PI3K-AKT-mTOR signaling pathway was found in early genetic sequencing studies [10]. TCGA consortium performed an integrated genome, transriptome, proteome, and methylome analysis of tumor specimens from 400 patients with ccRCC [4]. Mutation of PTEN, TSC1/2, and PIK3CA, which were detected in the early sequencing studies and all reside in the PI3K-AKT-mTOR pathway, could be confirmed by TCGA in 28 % of the tissues. Furthermore, they were the first to report comprehensive information on the RNA expression levels of various genes involved in metabolism (see Fig. 1 for an overview of the alterations). Fatty acid synthesis (FASN) and the glutamine- (SLC1A5) and lactate transporter genes (MCT1 and MCT4) were frequently found to be upregulated in aggressive tumors. Hexokinase, phosphofructokinase (PFK), pyruvate dehydrogenase kinase (PDK), alpha-enolase (ENO1), and lactate dehydrogenase (LDH-A) are critical enzymes in the glycolytic reactions and also examples of upregulated genes in TCGA RNA expression analysis [4]. Overexpression of LDH-A, MCT4, and ENO1 has been confirmed in independent patient cohorts [11–14]. The pentose phosphate pathway (PPP) branches from the glycolytic pathway and also appeared to be activated. Glucose-6-phosphate dehydrogenase (G6PD) and transketolase (TKT) are bidirectional and rate-limiting enzymes in the PPP with elevated expression levels [4]. Aconitase 2 (ACO2), α-ketoglutarate dehydrogenase (OGDH), and succinate dehydrogenase (SDHB) are examples of enzymes in the TCA cycle with diminished expression in TCGA RNA expression analysis [4]. Reduced AMP-activated kinase (AMPK) and increased acetyl-CoA carboxylase (ACC) protein levels were observed with a notable independent association with poor overall survival of patients [4].

Gene expression profiles have also been analyzed through mass spectrometry [15]. This analysis revealed upregulation of protein levels of glyceraldehyde-phosphate-dehydrogenase (GAPDH), phosphoglycerate kinase (PGK1), ENO2, pyruvate kinase (PKM2), and LDH-A in RCC specimens (*n* = 40) as compared to normal tissue [15]. The TCA cycle enzymes pyruvate carboxylase (PC), SDHA, and SDHB were downregulated. The same pattern was observed for enzymes involved the β-oxidation of fatty acids like hydroxylacyl-CoA dehydrogenase (HADH), short-chain enoyl-coA hydratase (SCEH), very long chain acyl-coenzyme A dehydrogenase (VLCAD), acetyl-coA acetyltransferase (ACAT1), and medium-chain acyl-coA dehydrogenase (MCAD). No significant differences in isocitrate dehydrogenase (IDH1/2) protein levels were observed between RCC and normal kidney tissues.

Regulation of glycolysis, amino acid metabolism, and lipogenesis has also been subject of two metabolomics studies performed on a single cohort of 138 patients with RCC [16, 17]. Real-time flux analysis provides superior information on upregulation of metabolic pathways as compared to the snapshot analysis performed in these two studies. Both up- and downregulation of individual metabolites can be indicative of increased metabolism, which hampers the interpretation of the results. Both Wettersten et al. and Hakimi et al. showed increased glucose, glu-6-P, fru-6-P, and lactate levels in RCC tissue as compared to healthy control tissue with notable association with the histopathologic nuclear grading system according to Fuhrman. Lower levels of TCA intermediates malate and fumarate were detected, while citrate and succinate were elevated. The levels of free fatty acids, glutamine, glutathione (GSH), and glutathione disulfide (GSSG) were increased and displayed an association with Fuhrman grade [16, 17]. Elevations of numerous consecutive metabolites (glucose, glu-6-p, fru-6-p, 6-PG) and their corresponding enzymes (HK, G6PD) do suggest upregulation of the first steps of the glycolytic pathway.

Certain aspects of these metabolomics studies require further investigation. Integrated analyses of transcriptomics and metabolomics showed a lack of linearity. For example, a remarkable depletion of a number of glycolytic metabolites (3-PG, 2-PG, and PEP) was noted along with overexpression of their corresponding glycolytic enzymes (PGK1, ENO2, PKM; see also Fig. 1) [17]. These results may reflect the fact that metabolic flux patterns do not always follow canonical flow but link and shunt into neighboring pathways. They indicate that more dynamic flux analyses are required to improve our understanding of this aspect of cancer biology. Moreover, they stress the importance of functional interference studies for development of therapeutics. The association between metabolites and Fuhrman grade does indicate that the natural disease course in patients with RCC, as reflected by increasing Fuhrman grade, is accompanied by progressive metabolic reprogramming.

The most recent metabolomics study marked shunting of glucose in the PPP and production of glutathione as poor prognostic features [17]. This conclusion is in agreement with the conclusion of the analysis by TCGA. Elevated levels of glucose corresponded to increases in the PPP intermediate 6-phosphogluconate (6-PG), supporting elevated PPP-flux. In addition, higher levels of GSH were detected along with its precursor glutamine in the poor prognosis subgroup. GSH buffers reactive oxygen species by serving as electron donor, which is accompanied by oxidation to GSSG. GSSG can be converted back to GSH by glutathione reductase. This reaction requires NADPH, which is an important product of G6PD activity in the PPP. Through this role, the PPP is thought to contribute to an improved redox state of RCC cells. High levels of GSH coincided with hotspot mutations in the gene encoding NRF2 and its negative regulator KEAP1. NRF2 is known to drive expression of multiple components of antioxidant defense system, including the GSH-producing enzymes [18]. Late stage RCC tumors may therefore increase the capacity to buffer oxidative stress, not only through upregulation of the PPP but also through activation of the NRF2 antioxidant response pathway.

### Molecular drivers of the metabolic reprogramming in RCC

Genome-wide association studies suggest a role of chromosome 3p loss in the metabolic reprogramming in RCC. The VHL, along with the metabolic regulator pyruvate dehydrogenase B (PDH-B), resides on a 50-Mb stretch of DNA on this chromosome. A large chromosomal copy number analysis indicated that this region is the most frequently deleted region in RCC (91 % of the samples) [19]. Loss of chromosome 3p and VHL correlated with divergence of metabolic gene expression in a large meta-analysis [5]. PBRM1, SETD2, and BAP1 are chromatin-modifying genes also located at chromosome 3p, but until now, no role has been identified for these genes in regulation of metabolism.

Transcriptional activity of the HIF may be the driving force behind the Warburg effect in RCC. Two isoforms of HIF exist, with the majority of RCC tumors (61 %) expressing both isoforms [20]. Loss of VHL results in normoxic HIF stabilization and enhances transcription of target genes. HIF1 was found to drive expression of GLUT1, PGK1, LDH-A, PDK1, and HK2 in transgenic mice with differential HIF1/HIF2 expression [21]. Suppression of oxidative phosphorylation in the TCA cycle was also found to be mediated by HIF1, through its target gene Dec1 and the transcriptional coactivator PGC-1α [22]. While evidence supports an important role of HIF1 in promoting aerobic glycolysis [21], it has also been shown to act as a tumor suppressor in preclinical RCC models [23]. Indeed, some tumors appear to have lost the HIF1 locus on chromosome 14q23, but surprisingly, no correlation was found between this deletion and HIF1A protein expression in RCC samples [19]. Future research will have to show whether correlations exist between HIF1 and/or HIF2 and the metabolic phenotype of RCC tumors.

Although loss of VHL may be a crucial event in RCC tumorigenesis, isolated loss does not elicit the characteristic HIF-transcriptional signatures [24]. Loss of fructose-1,6-bisphosphatase 1 (FBP1) on chromosome 9q22 is another unique feature of RCC [19]. Examination of its expression in 600 human RCC specimens revealed depletion in almost 100 % of the tumors as compared to normal kidney tissue [25]. Manipulation of FBP1 in normal kidney cells recapitulated numerous features of ccRCC tumors with markedly elevated glucose consumption and increased production of lactate, GSH, and NADPH [25]. Under physiological conditions, FBP1 opposes glycolysis and supports gluconeogenesis through production of Fru-6-p from Fru-1,6-P2 (see Fig. 1). In addition to this role in gluconeogenesis, the molecule was found to interact directly with HIF1 and HIF2, restraining their transcriptional activity [25]. Loss of FBP1 was associated with expression of several HIF-target genes, including PDK1, LDH-A, and GLUT1 [25]. Combined loss of VHL and FBP1 may therefore be an essential step in the development of the aerobic glycolysis phenotype in RCC.

The previously described molecular profiling studies revealed high levels of glutamine and free fatty acids in RCC specimens. Preclinical research indicates that these upregulated levels may be related to each other. In addition to its function as GSH precursor, glutamine can be transformed into α-ketoglutarate and oxidized in the TCA cycle. OGDH, which is the enzyme responsible for metabolism of α-ketoglutarate in the TCA cycle, was frequently found to be downregulated in RCC cells. IDH diverts α-ketoglutarate in the opposite direction in the TCA cycle through a process called “reductive carboxylation” [26]. Glutamine is transformed into citrate and acetyl-CoA in this pathway (Fig. 1) [27]. ACC and FASN mediate subsequent formation of fatty acids. The elevated levels of the ACC and FASN gene products as well as glutamine, citrate, and fatty acids in RCC specimens support potential activation of this pathway. High levels of fatty acids were measured in RCC cells, along with downregulation of the enzymes involved in β-oxidation [15]. Histological analysis showed intracellular storage of fatty acids as “lipid droplets” near the ER [7]. Storage of lipids relied on the HIF2-dependent gene perilipin 2 (PLIN2) and suppressed cytotoxic ER stress through a reduction of protein synthesis [7]. This information shows that tumor cells may upregulate glutamine-dependent lipogenesis to reduce ER stress.

Dual activation of HIF2 and MYC seems to induce glutamine-dependent lipogenesis. Focal amplification was found of chromosome 8q24 in RCC specimens, which is the locus of the transcription factor MYC [4]. Overexpression of MYC in transgenic mouse models of RCC induced upregulation of glutaminases (GLS1-2) and transporters (SLC1A5) and elevated levels of glutamate and α-ketoglutarate [28]. Upregulation of glutamine metabolism was also accompanied by accumulation of lipids in RCC tumors [28]. In addition to MYC, a role has been identified for HIF2 in glutamine-dependent lipogenesis [29]. Expression of a constitutively active HIF2 molecule followed by metabolic flux analysis, revealed a shift of IDH/ACO towards reductive carboxylation of glutamine to citrate [29]. Experiments with labeled glutamine isotopes showed increased production of lipogenic acetyl-coA upon expression of this active HIF2 molecule [29]. Furthermore, HIF2 is known to enhance transcriptional activity of MYC directly through increased promotor binding [30]. Altogether, this information implicates a concerted effort of HIF2 and MYC in activation of glutamine-dependent lipogenesis.

**Fig. 2:**
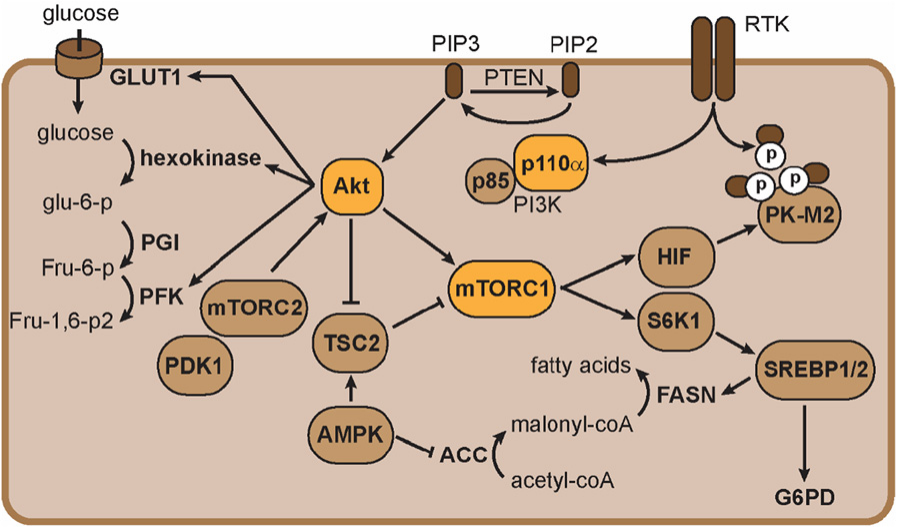
Connections between the PI3K- and metabolic pathways are shown. Given their central role in the pathogenesis of RCC, p110alpha, Akt, and mTORC1 are labeled *orange*

The activation of mTOR may also contribute to the metabolic phenotype of RCC tumors. Isolated activation of mTORC1 through TSC1/2 knock out induced a gene expression profile consistent with aerobic glycolysis [31]. Subsequent analysis of target genes combined with functional interference experiments revealed that HIF1 is responsible for the glycolytic response in this model [31]. Upregulation of HIF1 and HIF2 protein levels were thought to be induced by mTORC1-mediated inhibition of 4E-BP1 [31]. SREBP1/2 were other critical players that were found to act downstream of mTORC1. The latter transcription factors were found to drive the gene expression of G6PD and FASN [31] (Figs. 1 and 2).

Studies focusing on down- and upstream signaling partners of mTORC1 also claimed a role in regulation of metabolism. AKT is an example of an upstream signaling partner of mTORC1, which indirectly promotes its activity through inhibition of TSC2 [32]. Activity of AKT correlated with expression of glucose transporters (GLUT1), hexokinase (HK), and PFK1 in cancer cells (Fig. 2) [33–35]. Isolated overexpression of activated AKT stimulated aerobic glycolysis and rendered cells susceptible to glucose withdrawal [36]. AMPK on the other hand is a player that negatively affects mTORC1 signaling. It reduces mTORC1 activity through TSC2. Enhanced AMPK activity rescued cancer cells from AKT-dependent glucose addiction through activation of beta-oxidation of fatty acids [37]. These results suggest that the TSC2-mTORC1 axis is of importance for the impact of AKT on tumor metabolism. Decreased protein expression of AMPK and activating mutations in the PI3K-AKT-MTOR pathway are frequently observed in RCC tumors [4]. These results provide at least circumstantial evidence that mTORC1, although partly through HIF-mediated mechanisms, contributes to the metabolic phenotype of RCC tumors.

## Part II. Targeting metabolic reprogramming in preclinical models

### GLUT-1 and hexokinase inhibitors

Uptake of glucose is primarily mediated by the GLUT-1 transporter, which can be targeted by drugs. Selective blocking of this transporter by the drug STF-31 was found to impede glucose uptake and selectively inhibit growth of RCC tumors [38]. Fasentin and WZB117 are other GLUT1-inhibitors with comparable effects on intracellular glucose levels. WZB117 induced activation of the ATP-sensing enzyme AMPK in lung cancer xenografts, indicating that it induces metabolic stress [39]. A direct comparison of these drugs has not been performed.

Hexokinase commits glucose to the glycolytic pathway and has been target of treatment for several decades. Particularly, the chemically modified glucose analog 2-deoxy-d-glucose (2DG) is a drug with a long-standing history in cancer research. The additional hydroxyl-group in 2DG prohibits metabolism after phosphorylation by hexokinase. The chemical modification thereby induces accumulation of 2-deoxy-glucose-6-phosphate, which inhibits hexokinase activity. No recent results have been published on application of the drug in preclinical RCC models. The drug 3-bromopyruvate (3-BrPa) is a second drug that interferes with hexokinase activity. Four isoforms of hexokinase have been identified to date. Hexokinase 2 was found to associate with anion channels at mitochondria, which appears important for mitochondrial homeostasis [40]. While 2DG acts as substrate for hexokinase, 3-BrPa is believed to interfere directly with its enzymatic activity and covalently bind hexokinase 2, which disrupts its association with mitochondria [40]. MCT1 facilitates import of 3-BrPA and its expression is therefore of crucial importance for sensitivity to the drug [41]. Treatment with 3-BrPa markedly reduced ATP production and induced cell death in primary RCC cell lines [42]. Lonidamine (LND) is the third drug that interferes with hexokinase activity. Application depleted intracellular ATP levels through inhibition of hexokinase 2 (HK2) and induced accumulation of lactate in cells [43]. A comparison between 3-BrPA and LND in glioblastoma cell lines revealed a different mechanism of cell death [44]. Accumulation of lactate and the different cell death mechanism suggest additional effects of LND on cellular metabolism in addition to HK2 inhibition. LND has not been tested in preclinical RCC models.

### Inhibitors of the pentose phosphate pathway

**Fig. 3:**
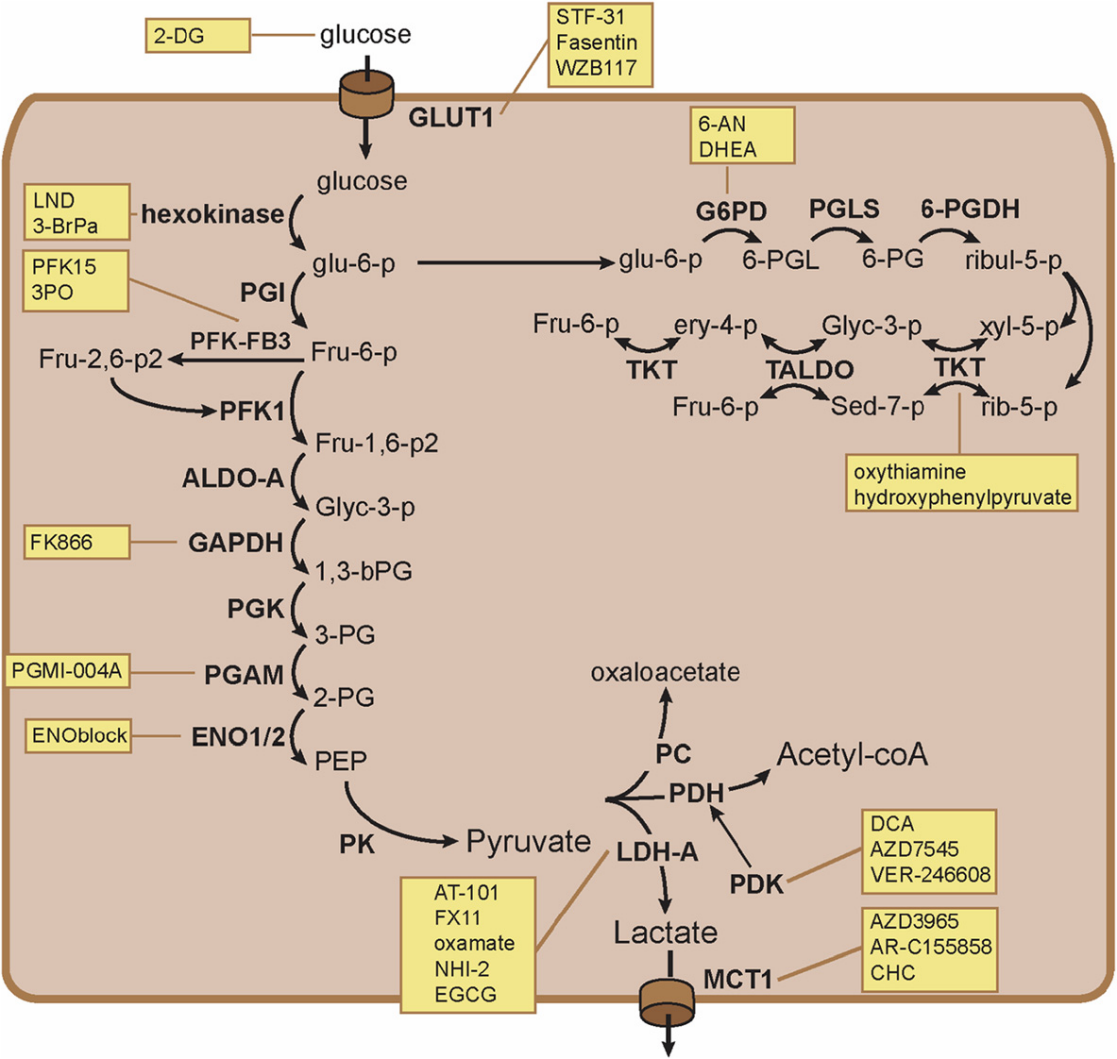
An overview of identified glycolytic inhibitors is shown along with their corresponding targets

G6PD and TKT are critical enzymes in the pentose phosphate pathway, whose activity can be blocked by drugs. Activity of hexokinase delivers glucose-6-phosphate which can be directed into the PPP (Fig. 3). The pathway consists of two distinct phases. G6PD is the rate-limiting enzyme in the oxidative phase, which generates NADPH and ribul-5-p. TKT acts as a critical enzyme in the second non-oxidative phase, which serves to recycle ribul-5-p back to the glycolysis through generation of Fru-6-p (Fig. 3). Silencing of TKT in hepatocellular carcinoma cells induced accumulation of ribul-5-p and rib-5-p [45]. Importantly, TKT and TALDO were found to function as bidirectional enzymes, having the ability to promote the reverse reaction. Depending on the metabolite balance in cancer cells, inhibition of TKT therefore may not necessarily reduce the production of Fru-6-p.

6-Aminonicotinamide (6-AN) and dehydroepiandrosteron (DHEA) are G6PD inhibitors that have been tested as experimental treatment. The drug 6-AN elevated oxidative stress in RCC cell lines through a decrease in NADPH [46], which is a known product of the PPP. Novel small molecule inhibitors with favorable pharmacokinetic properties are in development [47]. HDM2 antagonists are another class of drugs that may reduce G6PD activity. They induce accumulation of p53, which directly interacts with G6PD and blocks its function [48]. HDM2 antagonists such as MI319 have been tested in combination with sunitinib in RCC xenograft models and showed potent reduction of tumor growth [49]. Beneficial effects of HDM2 inhibitors could, however, also be mediated by other p53-dependent effects.

TKT is the second critical enzyme in the pentose phosphate pathway. Oxythiamine and hydroxyphenylpyruvate are examples of TKT inhibitors. Oxythiamine was found to be an effective treatment in hepatocellular carcinoma xenografts. Here, it reduced tumor growth through elevation of oxidative stress [45]. Treatment of Ehrlich’s ascitic tumor cells with oxythiamine significantly reduced the synthesis of ribose, which is a main product of the PPP and an important source for nucleic acid catabolism [50]. A variant of this thiamine analog has been generated with improved TKT specificity, but requires further investigation in preclinical models [51].

### Inhibitors of glycolytic enzymes

PFK1 catalyzes the production of fructose-1,6-bisphosphate (Fru-1,6-P2) from fru-6-p. The bidirectional enzyme PFK2-FBase regulates the production of fructose-2,6-bisphosphate (Fru-2,6-P2) from fru-6-p. Fru-2,6-P2 stimulates glycolysis through allosteric activation of PFK1 (Fig. 3). Four different isoforms of PFK2-FBase are known to be expressed in human tissues. The PFK-FB3 isoform was found to be preferentially expressed in cancer cells. The drug PFK15, as well as its predecessor 3PO, interferes with the enzymatic function of the kinase. As a consequence of treatment, reduced intracellular Fru-2,6-P2, lactate and ATP levels were detected in leukemic-, lung-, and breast cancer cells, which confirms on-target effects [52]. Treatment also induced potent antitumor effects in multiple xenograft models of cancer [53].

Fructose-bisphosphate-aldolase, GAPDH, PGK, phosphoglycerate mutase (PGAM1), and ENO1 are other enzymes involved in glycolytic reactions (Fig. 3). Small molecule inhibitors of GAPDH (FK866), PGAM1 (PGMI-004A), and ENO (ENOblock) have been discovered [54–56]. Pharmacological blockade of PGAM1 reduced glycolysis and resulted in accumulation of 3-phosphoglycerate (3-PG). Unexpectedly, PGAM1 inhibitors activated a feedback loop that reduced 6-phosphogluconate dehydrogenase (6-PGDH) activity and flux through the PPP [56]. A shift in the melting temperature of 6-PGDH was found in the thermal melt assay upon incubation with increasing concentrations of 3-PG. Further crystal structure analysis confirmed direct binding of 3-PG to 6-PGDH at sites important for enzymatic activity. Blockade of the PPP at the level of 6-PGDH through a toxic buildup of 3-PG therefore seems a mechanism for this activity [56].

PK catalyzes the final reaction in glycolysis and therapeutic strategies have been developed that target PK. In normal cells, PK generates ATP and pyruvate through dephosphorylation of phosphoenolpyruvic acid (PEP). The M2 isoform (PKM2) was shown to be preferentially expressed in cancer cells and promote aerobic glycolysis [57]. Interestingly, it was not its enzymatic activity but rather its ability to bind phospho-tyrosine proteins that was shown to promote aerobic glycolysis (Fig. 2) [58, 59]. Expression of PKM2 protein was found to be increased in human RCC specimens [15]. A systematic search for small molecules that selectively target PKM2 yielded numerous compounds that mimicked gene silencing in lung cancer cells [60]. Activation of enzymatic activity was also shown to serve as potential therapeutic strategy. PKM2 activity is known to be stimulated by upstream Fru-1,6-P2 through binding to a unique domain, which induces a conformational change. Compounds have been developed that mimick Fru-1,6-P2 activity in lung cancer cells and lock PKM2 in its active state [61]. Activation of PKM2 was shown to create a unique dependence of cancer cells on extracellular serine, most likely through reduced availability of 3-PG [61]. Tests of PK-M2 targeting strategies in xenografts and preclinical RCC models are lacking, and therefore, their role in this disease still has to be determined.

### Inhibitors of pyruvate and lactate metabolism

PDH converts pyruvate into acetyl-coA, while LDH transforms it into lactate. PDK inactivates PDH through its phosphorylation. Therapeutic strategies that interfere with pyruvate metabolism have focused on PDK in order to promote use of the TCA cycle. Downregulation of PDK both increased respiration and oxidative stress through activation of the TCA cycle [62]. Dichloroacetic acid (DCA) is an example of a PDK inhibitor that acts as pyruvate mimetic. DCA was found to reverse mitochondrial suppression and reduce tumor size of preclinical RCC xenografts [63]. AZD7545 and VER-246608 are other drugs that inactivate PDK through binding at different structural sites [64]. VER-246608 is an ATP-competitive PDK inhibitor, which enhanced PDH activity and cellular respiration and attenuated glycolytic activity in cancer cell lines [65].

LDH catalyzes the final step of aerobic glycolysis and represents another attractive target for treatment (Fig. 3). Similar to PDK inhibitors, downregulation of LDH-A was found to promote respiration in FH-deficient RCC cells [66]. At least two small molecule inhibitors have been tested in preclinical models. AT-101 or gossypol is an unspecific inhibitor of multiple dehydrogenase enzymes, including LDH-A. Its derivative FX11 is an example of a LDH-inhibiting compound, that was successfully tested in RCC models [67]. Treatment increased oxygen consumption and oxidative stress, indicating that pyruvate is shunted into the TCA cycle. Oxamate represents a second example of a promising LDH-inhibitor. Treatment induced similar metabolic results as found with FX11 with additional activation of autophagy in NSCLC cells [68]. *N*-hydroxyindole-2-carboxylates (NHI) are currently under investigation as next-generation LDH inhibitor. Epigallocatechin gallate (EGCG) may also have activity as LDH inhibitor but was found to interfere with other metabolic enzymes. Its utility as LDH inhibitor remains to be shown.

**Fig. 4:**
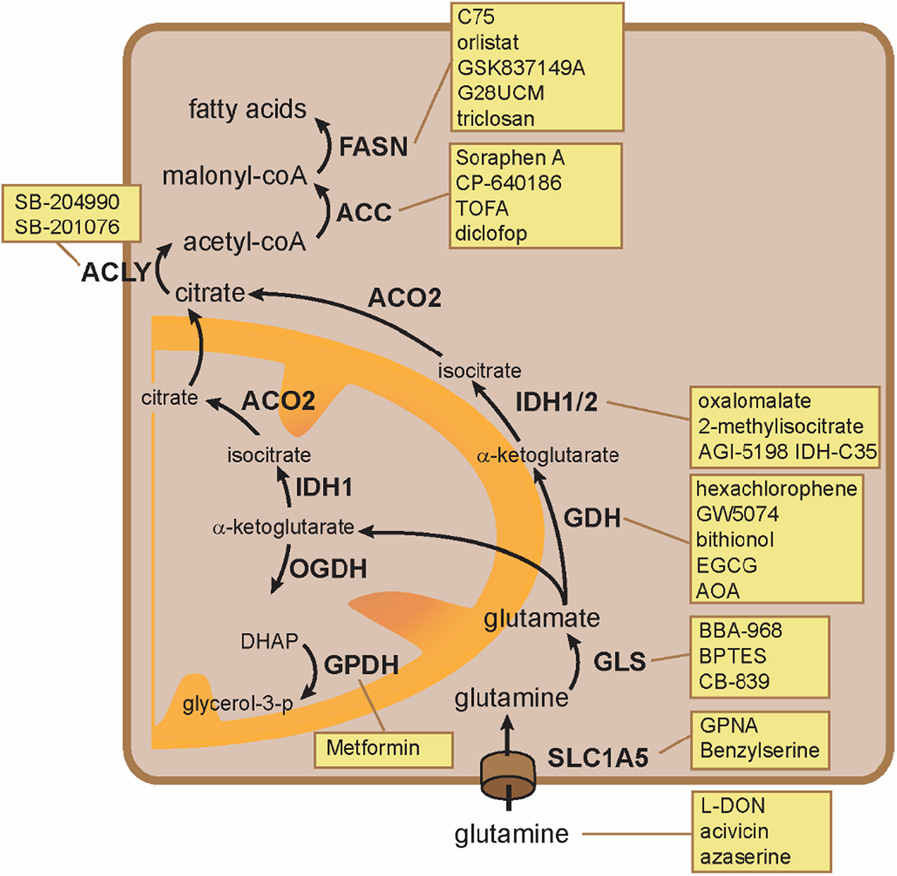
A schematic overview of drugs that may interfere with glutamine-dependent lipogenesis

Under physiological circumstances, lactate, as well as glycerol, can be employed for gluconeogenesis. The mitochondrial enzyme glycerophosphate dehydrogenase (GPDH) converts glycerol-3-phosphate (glyc-3-p) into dihydroxyacetone phosphate (DHAP) (Fig. 4). This reaction yields significant amounts of NAD+, which is required for the production of glucose from lactate. The antidiabetic drug metformin was found to block gluconeogenesis through specific targeting of the mitochondrial isoform of GPDH [69]. Secondary to these effects, AMPK activation was found in cancer cells treated with metformin, restraining mTORC1 signaling and limiting growth of renal cell cancer xenografts [70]. The importance of a functional AMPK-TSC1-mTORC1 pathway for metformin has been illustrated by the lack of response in a TSC1(+/−) transgenic model [71]. Frequent downregulation of AMPK has been observed in RCC and therefore makes the potential of the drug in RCC questionable.

Inhibition of lactate transport has been investigated as therapeutic strategy. The monocarboxylate transporters 1 and 4 (MCT1/4) facilitate lactate export from cancer cells and prevent a toxic buildup of the metabolite (Fig. 3). Downregulation of the MCT4 (SLC16A3) transporter was identified as therapeutic strategy through whole genome siRNA screens in RCC cell lines [72]. Lactate export is accompanied by H (+) transport across the plasma membrane. Through interference with intracellular pH, rapid cytotoxic effects are induced. AZD3965, a-cyano-4-hydroxy-cinnamate (CHC), and AR-C155858 are examples of drugs that interfere with lactate export through blockade of MCT1. AZD3965 treatment effectively induced lactate accumulation in small cell lung cancer xenografts [73]. AR-C155858 and CHC curtailed lactate secretion along with lower pH levels and cytotoxic effects in multiple myeloma cells [74]. Effectivity of MCT1 inhibitors appears to rely on low MCT4 expression levels [75], which suggest some functional redundancy. Both transporters seem to be expressed in RCC [13, 72]. Interestingly, silencing of MCT4 expression alone did affect the viability of RCC cells [72]. Future research will therefore have to focus on development of MCT4 or potential dual MCT1/4 inhibitors for patients with RCC.

### Arginine- and other amino acid-depleting treatment strategies

Metabolomics studies revealed decreased levels of almost all amino acids (except cysteine, glutamate, and glutamine) in RCC tumors as compared to normal kidney tissues [17]. In contrast, upregulation of genes involved in nucleotide and particularly pyrimidine synthesis was found in a meta-analysis of TCGA data [5]. Glutamine acts as source for nucleotide biosynthesis through conversion into TCA cycle intermediates [76]. ASS1 is an example of a gene involved in both amino acid and nucleotide metabolism. Downregulation of ASS1 has been detected in an independent analysis of RCC specimens [8]. ASS1 and downstream argininosuccinate lyase catalyze the conversion of aspartate into arginine. Lack of ASS1 results in accumulation of aspartate in the cytosol, which is subsequently directed into the pyrimidine and nucleotide synthesis pathway [77]. Downregulation of ASS1 therefore promotes generation of nucleotide precursors and also makes RCC cells dependent on extracellular arginine.

Depletion of extracellular arginine, through administration of pegylated arginase 1 (Peg-Arg-1) or arginine deiminase (ADI), has been explored as an anticancer treatment. It has been tested in RCC xenograft models as therapeutic strategy [8]. Dose-dependent inhibition of tumor growth was observed. Reconstitution of ASS1 induced resistance to ADI in vitro. Similar treatment effects were seen in hepatocellular carcinoma (HCC) and melanoma xenografts, with a sustained decrease in intratumoral arginine levels [78]. Unfortunately, many immune cells rely on extracellular arginine [79]. Arginine depletion therapy therefore has immunosuppressive effects, which possibly limits its efficacy in mice.

Tumor cells meet their essential amino acid demands by upregulation of specific transporters. The L-type amino acid transporter 1 (LAT1) is an example of an upregulated transporter in ccRCC [80]. It transports large neutral branched amino acids, such as the leucine and tryptophan. LAT1 recognizes amine and carboxylic acid side groups in substrates, which determines its selectivity. Drugs (JPH203, 3,5-diiodo-l-tyrosine, acivicin) have been developed that exploit this feature and competitively inhibit LAT1 activity. Treatment with JPH203 induced dramatic inhibition of leucine uptake and proliferation of colorectal cancer cells [81].

In line with the elevated expression of LAT1, increased tryptophan metabolism has been detected in RCC specimens [16]. Particularly, upregulation of the metabolic enzyme indoleamine 2,3-dioxygenase (IDO1) has been observed as a frequent feature [82]. This enzyme stimulates conversion of tryptophan into the immunosuppressive metabolite kynurenine [83], which was also found to be elevated in RCC [16]. The IDO-inhibitors L-1-MT, 1-D-MT, INCB024360, and indoximod have been developed and give various degrees of IDO-blockade. A decrease in kynurenine production was found in vitro [16, 84]. Effectivity of these inhibitors has not been tested in RCC xenograft models.

### Inhibitors of glutamine metabolism

Glutamine analogs as well as inhibitors of physiological glutamine import have been used as therapeutic agents in preclinical studies. In addition to its function as an energy and lipid source, glutamine participates in many enzymatic reactions. The glutamine analogs 6-diazo-5-oxo-L-norleucine (L-DON, NSC 7365), acivicin (NSC 163501), and azaserine (NSC 742) block the function of glutamine utilizing enzymes (Fig. 4). Pharmacological in vitro studies using leukemia cells identified inhibition of amidotransferases and nucleotide biosynthesis as principal mechanism of action [85]. Import of glutamine into cancer cells is exerted by SLC1A5 (ASCT2). Gamma-l-glutamyl-*p*-nitroanilide (GPNA) and benzylserine (H-Ser(Bzl)-OH) are targeted inhibitors of this transporter that reduce growth of lung cancer xenografts. GPNA treatment of lung and prostate cancer cells diminished glutamine consumption and generation of fatty acids and induced autophagy, suggesting that it causes metabolic stress [86, 87]. None of these inhibitors has been tested in preclinical RCC models.

Glutaminase (GLS) is the enzyme involved in the conversion of glutamine into glutamate (Fig. 4). It can be targeted with the chemical inhibitors bis-2-(5-phenylacetamido-1,2,4-thiadiazol-2-yl)ethyl sulfide (BPTES), bromo-benzophenanthridinone 968 and CB-839. The inhibitor BPTES has been successfully tested in preclinical RCC models. It suppressed growth of RCC xenografts in two independent studies [28, 29]. Application of BPTES in glioblastoma cell lines reduced glutaminase activity with reduced glutamate and α-ketoglutarate production [88]. Similar results have been obtained with CB-839 in breast cancer cells [89].

Glutamate dehydrogenase (GDH) converts glutamate into α-ketoglutarate (Fig. 4). Hexachlorophene, GW5074 and bithionol are GDH-inhibitors. Their structure was based on the green tea polyphenol epigallocatechin gallate (EGCG). This drug has frequently been used in preclinical experiments as GDH-inhibitor [90, 91], although it may have a variety of other effects in cancer cells. Amino oxyacetate (AOA) is a well-characterized inhibitor of transaminases [92]. AOA was shown to affect glutamate metabolism via a pathway involving an aminotransferase, presumably aspartate aminotransferase [93]. Both AOA and EGCG effects were shown to be rescued by supplementation of molecules such as α-ketoglutarate or oxaloacetate, supporting specific effects on glutamine metabolism [91, 94].

Oxalomalate and 2-methylisocitrate are NADPH-competitive IDH inhibitors. Reductive carboxylation of α-ketoglutarate by IDH was shown to be the dominant pathway for generation of citrate and lipids in RCC cells [27, 95]. Both oxalomalate and 2-methylisocitrate reduced integration of isotope-labeled glutamine into citrate and lipids in adipocytes [96]. Discovery of IDH1 mutations in certain diseases urged development of mutant specific inhibitors such as AGI-5198 IDH C-35, AG120, and AG221. Limited affinity for wild-type IDH1/2, such as observed in RCC, was found, leaving little room for application in this disease [97]. In addition to citrate, 2-hydroxyglutarate (2-HG) is produced during reductive carboxylation of α-ketoglutarate [95]. Accumulation of 2-HG was found in RCC cells, which was mainly attributed to decreased expression of the degrading enzyme 2-HG dehydrogenase (L2HGDH) [98]. No therapeutic strategies have been developed to target this last enzymatic process.

### Inhibitors of lipogenesis

Citrate can be transported out of the mitochondria and cleaved to acetyl-coA by ATP citrate lyase (ACLY). Activity of ACLY can be blocked by the chemical inhibitors SB-204990 and SB-201076. The depletion of ACLY inhibits proliferation of lung-, liver-, and prostate cancer cells under lipid-deprived growth conditions, supporting a crucial role in lipogenesis [99]. Significant reductions in acetyl-coA levels were found in cancer cells after treatment with the chemical ACLY inhibitor SB-204990 [100]. Single agent activity was shown in lung and prostate cancer xenograft models with predominantly cytostatic effects [100]. No results have been published with these agents in RCC models.

ACC carboxylates acetyl-coA to form malonyl-coA. Its expression was independently associated with worse clinical outcome of patients with RCC. Three chemical inhibitors of ACC have been tested as anticancer treatment. Downregulation of ACC through RNA interference or with the chemical inhibitor soraphen A attenuated fatty acid synthesis and proliferation of prostate cancer cells [101]. The cytotoxic effects were abolished by supplementation of culture media with fatty acids, which supports interference with lipid metabolism as the principal mechanism of action. CP-640186 and TOFA are the other two chemical inhibitors that have been tested in preclinical liver and breast cancer models [9, 102]. Treatment of breast cancer cells with TOFA reduced malonyl-coA levels [103], confirming on-target effects of this drug.

FASN converts malonyl-coA to long-chain fatty acids. Inhibition of FASN by cerulin or its derivative C75 induced a rapid increase in malonyl-coA with a marked reduction in lipogenesis in multiple cancer models, including RCC cells [103, 104]. In addition to effects on lipogenesis, accumulation of malonyl-coA may have cytotoxic effects in itself. Orlistat, GSK 837149A, G28UCM, and triclosan are other FASN inhibitors that have shown to limit proliferation of prostate and ovarian cancer cells [105–107]. Consistent with inhibition of de novo fatty acid synthesis, G28UCM and C75 treatment caused conversion of membrane lipids into storage lipids in ovarian cancer cells [107]. A reduction in overall lipid content was found after triclosan and C75 treatment in prostate cancer cells [105].

### Inhibitors of the PI3K-AKT-mTOR pathway

Due to the abundance of molecular alterations in the PI3K-AKT-mTOR pathway in RCC, several therapeutic strategies have been explored. Many of the inhibitors described below exhibit cross-activity against other components of the pathway (for example, dual PI3K/mTOR inhibitors). More information about the pharmacological aspects of these drugs can be found elsewhere. Activating mutations in p110 and p85 subunits of PI3K as well as inactivating mutations in the PTEN phosphatase presumably render tumors susceptible to targeted inhibitors. Indeed, promising results have been achieved with PI3K-inhibitors such as NVP-BEZ235, GDC-0980, and LY294002 in renal cell cancer models [108–112]. Perifosine (KRX-0401) is an example of an AKT inhibitor that has been described to reduce proliferation of RCC cells [113]. Clinical activity of rapalogs (temsirolimus, everolimus) in patients with RCC elicited development of next-generation mTOR inhibitors. Particularly enhanced activity against mTORC2 seems to improve efficacy and these agents are therefore awaiting clinical tests. WYE-125132, WYE-354, P7170, and AZD8055 are examples of novel mTOR inhibitors that induced tumor shrinkage in preclinical RCC models [114–117].

## Part III. Clinical trials on metabolic inhibitors

### Clinical experience with metabolic inhibitors

Many of the drugs that were tested in early stages of drug development in oncology were targeting metabolic components of tumors. New insights from molecular profiling of numerous diseases and preclinical experiments may urge a revival of drugs from this era. Although these drugs have been tested in the clinic, it remains uncertain whether dosing, treatment schedule, tumor type, and response evaluation in these trials were adequate. In the ideal situation, one prefers to evaluate on-target effects in patient-derived tumor tissue. This last action may differentiate between suboptimal dosing regimens, pharmacologically inferior drugs, and unviable drug targets. From common genetic polymorphisms that are known to confer a clinical phenotype, such as the single nucleotide polymorphisms (SNP) in the G6PD gene that may cause severe hemolytic anemia, one may anticipate toxicity of certain inhibitors due to on-target effects. These on-target effects on non-tumor tissue are unwanted during treatment but can be very informative for evaluation of pharmacodynamic effects. Occurrence of certain side effects (e.g., hemolytic anemia in G6PD blockade) in early clinical trials may tell whether the investigators reached proper blockade of the target during the treatment. Both efficacy and toxicity may therefore provide valuable information for design of clinical trials with these agents or their improved counterparts.

The glucose analog 2-deoxy-d-glucose (2DG) is an example of a drug that has received renewed attention lately (see Table 1 for an overview). A traditional dose escalation study was performed among patients with advanced cancer as single agent as well as in combination with docetaxel [118, 119]. On a daily dosing schedule of 45–60 mg/kg, plasma concentrations of 0.7 mM were reached which approximates concentrations used for preclinical RCC experiments [118, 120]. Dose-limiting toxicities (DLTs) were fatigue, sweating, dizziness, nausea, and prolonged QTc time. These results suggest that 2DG may induce hypoglycemic symptoms due to competition with systemic glucose. PET imaging in a limited number of patients showed reduced uptake of fluoro-deoxy glucose (FDG), indicative of potential competition between FDG and 2DG in tumors. No phase 2 study has been performed with this drug in RCC.

**Table 1:** Overview of drugs, currently in clinical development

Drug	Target	Clinical stage	RCC
2DG	Glucose	Phase 2	n.a.
Lonidamine	HK	Phase 2	Negative
Dichloroacetate (DCA)	PDK	Phase 3	n.a.
Polyphenon E	LDH-A	Phase 1	n.a.
FK866	GAPDH	Phase 1	n.a.
AT-101	LDH-A	Phase 2	n.a.
PEG-ADI	Arginase 1	Phase 3	n.a.
Indoximod	LAT1	Phase 2	n.a.
Acivicin	Glutamine	Phase 2	Negative
6-Diazo-5-oxo-l-norleucine (DON)	Glutamine	Phase 2	n.a.
Orlistat	FASN	Phase 1	n.a.
Metformin	GPDH	Phase 3	n.a.
NVP-BEZ235	PI3K/mTOR	Phase 2	n.a.
GDC-0980 (apitolisib)	PI3K/mTOR	Phase 2	n.a.
SF1126	PI3K	Phase 1	n.a.
BYL719	P110a	Phase 2	n.a.
MLN1117	P110a	Phase 2	n.a.
AZD8186	p110β	Phase 1	n.a.
GSK2636771	p110β	Phase 2	n.a.
SAR260301	p110β	Phase 1	n.a.
Perifosine	Akt	Phase 2	Negative
MK-2206	Akt	Phase 2	n.a.
GSK690693	Akt	Phase 1	n.a.
GDC-0068	Akt	Phase 2	n.a.
BEZ235	PI3K/mTOR	Phase 2	n.a.
XL765	PI3K/mTOR	Phase 1	n.a.
GDC0890	PI3K/mTOR	Phase 2	n.a.
GSK1059615	PI3K/mTOR	Phase 1	n.a.
AZD8055	mTORC1/2	Phase 1	n.a.
AZD2014	mTORC1/2	Phase 2	Negative

Lonidamine is another revived chemotherapeutic agent, which is supposed to target hexokinase in tumors. Phase 1 clinical trials have been conducted in the 1980s as single agent as well as combined with whole body hyperthermia [121–123]. Safety was confirmed of chronic use of oral doses up to 360 mg/m^2^, yielding systemic concentrations of 5.13 μg/mL. Side effects consisted mostly in musculoskeletal discomfort, anorexia, testicular pain in males, and a reversible ototoxicity. No metabolism or target inhibition analyses were performed in patients. Two phase 2 studies have been performed among patients with RCC, showing limited clinical activity of the drug [124, 125]. One year survival rate was 37.5 % in patients treated with lonidamine which relates unfavorable to current treatment strategies [124]. Limited information is available on RCC subtype and patient characteristics (e.g., Heng prognostic score) in these trials. The results therefore have to be interpreted with caution.

The small molecule PDK-inhibitor dichloroacetate (DCA) is registered for patients with congenitally dysfunctional PDH. Safety and tolerability has also been tested in patients with advanced solid tumors [126]. The maximum tolerated dose was 6.25 mg/kg with fatigue, nausea, and grade 3 neuropathy as dose limiting toxicity at higher dose levels. Pharmacokinetic analysis revealed high variability in systemic drug concentrations, possibly related to common genetic polymorphisms in the GSTZ1/MAAI metabolic enzyme [126, 127]. Preliminary analysis of glioblastoma tissue from three patients treated with DCA showed stimulation of PDH activity and accumulation of α-ketoglutarate and hydrogen peroxide [128]. Further research is warranted to determine efficacy of the drug as potential therapeutic agent.

Based on the cancer preventive as well as therapeutic activity of the multitargeted agent epigallocatechin gallate (EGCG), phase 1 clinical trials have been conducted. Most clinical trials used polyphenon E as a defined decaffeinated green tea polyphenol mixture. Doses up to 2000 mg twice daily were well tolerated with predominantly mild to moderate gastrointestinal side effects such as diarrhea and nausea [129, 130]. No dose-limiting toxicities or pharmacodynamic results have been reported. The oral bioavailability was shown to be poor with large variability and a median plasma through concentrations of 40.4 ng/mL [130]. Although uptake was shown to be improved by fasting prior to intake, concentrations relate unfavorable to preclinically effective levels (5–50 times higher) [131]. Alternative pharmacological prescriptions are warranted for intravenous testing and maximum tolerable dose (MTD) determination. Current polyphenon formulations are most likely insufficient for proper clinical trials in RCC.

Metformin is a commonly used antidiabetic drug that was found to have anticancer activity. Its favorable side-effect profile and abundant clinical experience has urged start of clinical trials. Beneficial effects of metformin doses up to 2000 mg per day have been observed on the regulation of diabetes mellitus. Retrospective cohort studies have confirmed potential anticancer activity of these doses in numerous patient cohorts. In a large renal cell cancer cohort, prolonged overall survival was seen in patients with localized disease but not in patients with metastatic disease [132]. Prospective phase 2 trials have been conducted, assuming maximum anticancer effects at commonly used antidiabetic doses. No prospective clinical trials have been done in RCC. Disease stabilization has been observed as best response among patients with prostate cancer, while no clinical activity was found in patients with pancreatic cancer [133, 134]. The correlation between AMPK expression and treatment response has not been subject of these analyses.

FK866 and AT-101 are two other drugs that have reached the early stages of clinical testing. AT-101 represents an unspecific inhibitor of dehydrogenases, including LDH-A. A phase I/II clinical trial among patients with prostate cancer showed an MTD of 20 mg/day [135]. Diarrhea, fatigue, nausea, and anorexia were the most frequently observed adverse events. The GAPDH and NAD-inhibitor FK866 was administered as a continuous intravenous infusion over 96 h. Thrombocytopenia was the dose limiting toxicity, yielding an MTD of 0.126 mg/m^2^/hour [136]. In addition, lymphopenia, anemia, and liver test abnormalities were observed. No metabolic analysis was performed during treatment with these inhibitors.

Pegylated arginine deiminase (PEG-ADI) and the IDO-inhibitor indoximod are drugs that underwent phase 1/2 clinical testing. Increasing doses of the arginine depleting enzyme PEG-ADI were given to 31 patients with melanoma with ASS1 negative tumors [137]. Pharmacodynamic analysis revealed neutralization of systemic arginine in 30/31 patients at day 8 after the start of treatment. No RECIST based responses were measured [137] with eight patients having stable disease as best response. No dose-limiting toxicities were seen during treatment. Indoximod has also been subject of a phase 1 clinical trial in 48 patients with advanced solid tumors [138]. The maximum tolerated dose was 2000 mg twice daily. Hypophisitis, tumor antigen autoantibodies, and elevated CRP levels were observed at several dose levels and suggest a reduction of immunosuppressive effects in tumors. Phase 2 clinical trials are underway.

Acivicin and 6-diazo-5-oxo-l-norleucine (DON) are glutamine antagonists that have been tested in patients with cancer. Acivicin and DON can both be isolated from *Streptomyces sviceus* bacterial cultures and are structurally related to glutamine. Dose escalation studies among patients with advanced cancer revealed MTDs of 600 mg/m2 for DON [139]. Multiple dosing regimens have been used for treatment with acivicin. Doses ranging from 15 mg/m^2^/day on five consecutive days to 160 mg/m^2^/day as single 24-h infusions were recommend by these studies [140, 141]. Acute dose-dependent nausea, vomiting, and diarrhea were DLTs after treatment with DON. Acivicin induced myelosuppression and neuropsychiatric symptoms (paresthesia, weakness, hallucinations, psychosis, confusion) as dose-limiting toxicities. These last symptoms were attributed to the potential neurotransmitter like properties of acivicin. Concomitant intravenous administration of amino acid mixtures was shown to reduce CNS toxicity and therefore allowed further dose escalation [142]. Pharmacodynamic analysis of amidotransferase acitivity in ascites derived tumor cells showed suppression by acivicin [140]. A randomized phase 2 trial has been performed with acivicin among patients with advanced RCC [143]. One partial response was observed after treatment with 20 mg/m^2^ for 72 h among 27 patients.

The FASN inhibitor orlistat is a drug that is approved for weight management in over 120 countries. A randomized double blind study among 539 obese individuals showed safety and good tolerability of the drug at a dose of 120 mg once daily [144]. However, negligible systemic absorption renders the oral drug unsuitable for treatment of patients with cancer [145]. Further research is needed to determine potential use of alternative pharmacological formulations of this drug.

### Clinical experience with PI3K-AKT-mTOR inhibitors

As downstream mediator of receptor tyrosine kinase (RTK) signaling as well as mutated hotspot itself, PI3K represents an attractive therapeutic target. A variety of inhibitors, including the previously described drugs NVP-BEZ235, GDC-0980, and SF1126, have entered clinical trials. PI3K targeting drugs can be divided in pan-PI3K- and isoform-specific PI3K inhibitors. Multiple pan-PI3K inhibitors underwent phase 1 and 2 clinical testing and showed limited toxicity and at best modest clinical activity [146]. Dose-limiting effects included hyperglycemia, maculopapular skin rash, and gastrointestinal intolerance (nausea, anorexia, diarrhea) [147–150]. AKT phosphorylation in blood, skin, or tumor tissue has been used as pharmacodynamic biomarker and showed a decrease, ranging from 40 to 90 %. FDG-PET imaging also showed marked metabolic responses in a small subset of patients [147]. Whether these effects are sufficient to induce durable treatment responses in patients with RCC is questionable. It has been conjectured that the essential role of PI3K in healthy tissues may limit dosing and restrict impact on tumors. Recent success with the δ-isoform-specific PI3K-inhibitor idelalisib in hematological malignancies [151] urged investigation of such specific inhibitors in solid tumors as strategy to circumvent these potential limitations of pan-PI3K inhibition. RCC tumors are known to harbor frequent PTEN and PIK3CA mutations. Previous research indicated that PTEN loss should be targeted by p110β-inhibitors [152], while PIK3CA mutations ask for p110α selective inhibitors [153]. The first clinical results of p110α selective (BYL719, MLN1117) and p110β-selective (AZD8186, GSK2636771, SAR260301) inhibitors are now emerging. Phase 2 clinical trials in patients with RCC will be required to further elucidate the role of these inhibitors in this disease.

AKT acts as critical downstream mediator of PI3K and has been postulated as “the Warburg kinase.” Perifosine and MK-2206 are examples of AKT inhibitors that were subject of phase 1 clinical trials [154, 155]. GSK690693 and GDC-0068 are ATP-competitive AKT inhibitors targeting all three isoforms that are currently under investigation. Dose-limiting toxicities were skin rash, nausea, diarrhea, pruritus, and hyperglycemia. AKT phosphorylation declined in tumor biopsies after treatment with MK-2206 [154]. Two phase 2 trials were conducted with perifosine among patients with RCC, showing limited clinical activity of the drug. No metabolic analysis has been performed in studies that employed AKT inhibitors. Preclinical studies suggested AKT-independent signaling pathways in PIK3CA mutated cell lines, possibly explaining limited clinical activity of perifosine [156].

Preclinical experiments suggest improved antitumor activity of dual targeting of PI3K/mTOR or mTORC1/mTORC2. Mutation of PIK3CA predisposes to a favorable response to rapalogs [157]. An elevated systemic LDH level before start of treatment was found to be associated to overall survival of patients with RCC treated with temsirolimus [158]. In both publications, the authors speculated that the biomarkers are indicative of pathway activation, which may explain their association with response to mTOR inhibitors. The drugs BEZ235, XL765, GDC-0890, and GSK1059615 are examples of dual PI3K/mTOR inhibitors. Phase 1 results from clinical tests with BEZ235 and XL765 have been published and showed similar toxicity profiles as with pan-PI3K inhibitors [159, 160]. AZD8055 and AZD2014 are examples of dual mTORC1/2 inhibitors that went through phase 1 testing [161, 162]. AZD2014 was shown to inhibit p-S6 in tumor biopsies. A randomized phase 2 trial has been conducted with AZD2014, comparing its efficacy with everolimus [163]. The clinical trial was stopped early due to inferior activity of AZD2014 in this patient cohort, despite adequate systemic drug concentrations. No results of pharmacodynamic analysis of tumor tissue were described.

### The impact of VEGF and mTOR inhibitors on tumor metabolism

Molecular profiling studies identified metabolic alterations as poor prognostic parameters. It has been suggested that upregulation of aerobic glycolysis and lipogenesis are part of the natural disease course and may aid in tumor progression. Most of these studies were performed in the era of targeted therapy, with VEGF-targeted drugs and mTOR inhibitors as established treatment modalities. Treatment poses selective pressure, allowing expansion of certain clones with advantageous molecular features. We here briefly review drug resistance against VEGF- and mTOR-targeted inhibitors and its role in directing tumor metabolism.

VEGF-targeted drugs are known to interfere with angiogenesis, significantly compromising oxygen and nutrient supply. Aerobic glycolysis seems an efficient way to cope with certain aspects of these types of metabolic stress, since it requires little oxygen. A comprehensive analysis of the impact of VEGF-targeted agents on tumor metabolism has been performed in lung and breast cancer xenografts. Upregulation of glycolysis and lipogenesis was found during treatment with the VEGF inhibitors sunitinib or sorafenib in this study [164]. Similarly, the VEGF-neutralizing antibody bevacizumab was shown to promote lactate production and activation of the PI3K-pathway in glioblastoma xenografts [165, 166]. Analysis of RCC tumor tissue after treatment with erlotinib and bevacizumab revealed an association between poor treatment response and low AMPK expression or activation of the PI3K-pathway. Sequential FDG-PET imaging during sunitinib treatment in patients with RCC showed high glucose uptake associated with poor treatment response [167]. These observations indicate that VEGF-targeted drugs may aid in selection of tumor cells with altered metabolism and/or activation of PI3K. Treatment with orlistat, DCA, and PI3K inhibitors restored the sensitivity to VEGF-targeted drugs in preclinical models, demonstrating potential of combination treatment strategies [164, 166, 168].

The rapalogs everolimus and temsirolimus interfere with mTOR signaling in tumor cells. Multiple resistance mechanisms have been identified in the last decade. These mechanisms include activation of MAPK-pathway through a PI3K-mediated feedback loop and increased survivin expression [169, 170]. TSC1/2 mutations were shown to predispose to a favorable treatment response [171]. Furthermore, mTOR acts as an energy sensor in cells and its inhibition leads to the activation of salvage pathways for the generation of energy, such as autophagy or utilization of extracellular amino acids [172, 173]. Functional interference with these salvage pathways can result in synthetic lethal effects in cancer cells. For example, severe cytotoxic effects of combined autophagy- and mTOR inhibition have been detected in mantle cell lymphoma cells [174]. Similarly, targeting compensatory upregulation of glutamine metabolism in glioblastoma xenografts induced severe cytotoxic effects [175]. The implications of “the metabolic shift” in RCC for the sensitivity to mTOR inhibitors, or the impact of mTOR inhibitors on metabolism are currently unknown and warrant further investigation.

## Conclusions

Molecular profiling studies have uncovered a pronounced metabolic shift in tumors of patients with ccRCC. Aerobic glycolysis, upregulation of lipogenesis and activation of the PI3K pathway are hallmarks of the disease. HIF1/2, MYC, and mTOR are deemed essential players in this feature. Preclinical research has revealed several promising therapeutic targets in these pathways, among them G6PD, PFK-FB3, and LDH-A. Several therapeutic agents showed promising pharmacodynamic effects and have been tested with success in preclinical models of RCC and are therefore awaiting clinical evaluation. Particularly, dual inhibitors of PGAM1 and 6-PGDH (e.g., PGMI-004A) and inhibitors of the relatively cancer-specific target PFK-FB3 such as PFK15 warrant further investigation. Disappointing results have been obtained in clinical trials with a number of drugs (e.g., orlistat, polyphenon E) due to a suboptimal pharmacological formulation. In other clinical studies a thorough pharmacodynamic analysis during treatment was lacking, limiting the possibility to draw conclusions on the viability of the drug targets. Novel PI3K-AKT-mTOR inhibitors have arrived in the clinic. Thus far, the results have however been disappointing, possibly due to a lack of tumor- or drug target-specificity. Future trials will rely on careful design, proper patient selection, and thorough analysis of the pharmacodynamic effects of drugs in order to shift paradigms and move towards new treatment regimens. To this end, combined molecular characterization before start and after treatment may be of critical importance for adequate treatment selection. Given the fact that metabolic remodeling in RCC carries a poor prognosis and aids in disease progression, therapeutic agents that interfere with this process may shape the future of patients with advanced RCC.

## Abbreviations

2DG, 2-deoxy-d-glucose; 3-BrPa, 3-bromopyruvate; 6-AN, 6-aminonicotinamide; 6-PG, 6-phosphogluconate; 6-PGDH, 6-phosphogluconate dehydrogenase; ACAT1, acetyl-coA acetyltransferase; ACC, acetyl-CoA carboxylase; ACLY, ATP citrate lyase; ACO2, aconitase 2; ADI, arginine deiminase; AMPK, AMP-activated kinase; AOA, amino oxyacetate; BPTES, bis-2-(5-phenylacetamido-1,2,4-thiadiazol-2-yl)ethyl sulfide; ccRCC, clear cell renal cell cancer; CHC, a-cyano-4-hydroxy-cinnamate; DCA, dichloroacetic acid; DHEA, dehydroepiandrosteron; EGCG, epigallocatechin gallate; ENO1, alpha-enolase; ER, endoplasmic reticulum; FASN, fatty acid synthesis; FDG, fluoro-deoxy glucose; Fru-2,6-P2, fructose-2,6-bisphosphate; G6PD, glucose-6-phosphate dehydrogenase; GAPDH, glyceraldehyde-phosphate-dehydrogenase; GLS1, glutaminase; GPNA, gamma-l-glutamyl-*p*-nitroanilide; GSH, glutathione; GSSG, glutathione disulfide; HADH, hydroxylacyl-CoA dehydrogenase; HCC, hepatocellular carcinoma; HIF, hypoxia inducible factors; HK, hexokinase; H-Ser(Bzl), OH benzylserine; IDH1, isocitrate dehydrogenase; L2HGDH, 2-HG dehydrogenase; LDH, lactate dehydrogenase; L-DON, 6-diazo-5-oxo-l-norleucine; LND, lonidamine; MCAD, medium-chain acyl-coA dehydrogenase; MCT, monocarboxylate transporters 1 and 4; MTD, maximum tolerable dose; NHI, *N*-hydroxyindole-2-carboxylates; OGDH, α-ketoglutarate dehydrogenase, oxoglutarate dehydrogenase; PDH, pyruvate dehydrogenase; PDK, pyruvate dehydrogenase kinase; PEG-ADI, pegylated arginine deiminase; PEP, phosphoenolpyruvic acid; PFK, phosphofructokinase; PGAM1, phosphoglycerate mutase; PGK1, phosphoglycerate kinase; PKM2, pyruvate kinase; PPP, pentose phosphate pathway; RTK, receptor tyrosine kinase; SCEH, short-chain enoyl-coA hydratase; SDHB, succinate dehydrogenase; TCA, tricarboxylic acid; TCGA, The Cancer Genome Atlas; TKT, transketolase; TOFA, 5-(tetradecycloxy)-2-furoic acid; VLCAD, very long chain acyl-coenzyme A dehydrogenase
